# Urine metabolomics links dysregulation of the tryptophan-kynurenine pathway to inflammation and severity of COVID-19

**DOI:** 10.1038/s41598-022-14292-w

**Published:** 2022-06-15

**Authors:** Joseph P. Dewulf, Manon Martin, Sandrine Marie, Fabie Oguz, Leila Belkhir, Julien De Greef, Jean Cyr Yombi, Xavier Wittebole, Pierre-François Laterre, Michel Jadoul, Laurent Gatto, Guido T. Bommer, Johann Morelle

**Affiliations:** 1 Laboratory of Inherited Metabolic Diseases/Biochemical Genetics, Cliniques universitaires Saint-Luc, Avenue Hippocrate 10, 1200 Brussels, Belgium; 2 Division of Nephrology, Cliniques universitaires Saint-Luc, Avenue Hippocrate 10, 1200 Brussels, Belgium; 3 Division of Infectious Diseases, Cliniques universitaires Saint-Luc, Brussels, Belgium; 4 Department of Intensive Care Medicine, Cliniques universitaires Saint-Luc, Brussels, Belgium; 5 Biochemistry, de Duve Institute, UCLouvain, Brussels, Belgium; 6 Computational Biology and Bioinformatics Unit (CBIO), de Duve Institute, UCLouvain, Brussels, Belgium; 7 Institut de Recherche Expérimentale et Clinique, UCLouvain, Brussels, Belgium

**Keywords:** Immunology, Biomarkers, Medical research, Nephrology

## Abstract

SARS-CoV-2 causes major disturbances in serum metabolite levels, associated with severity of the immune response. Despite the numerous advantages of urine for biomarker discovery, the potential association between urine metabolites and disease severity has not been investigated in coronavirus disease 2019 (COVID-19). In a proof-of-concept study, we performed quantitative urine metabolomics in patients hospitalized with COVID-19 and controls using LC–MS/MS. We assessed whether metabolites alterations were associated with COVID-19, disease severity, and inflammation. The study included 56 patients hospitalized with COVID-19 (26 non-critical and 30 critical disease); 16 healthy controls; and 3 controls with proximal tubule dysfunction unrelated to SARS-CoV-2. Metabolomic profiling revealed a major urinary increase of tryptophan metabolites kynurenine (*P* < 0.001), 3-hydroxykynurenine (*P* < 0.001) and 3-hydroxyanthranilate (*P* < 0.001) in SARS-CoV-2 infected patients. Urine levels of kynurenines were associated with disease severity and systemic inflammation (kynurenine, *r* 0.43, *P* = 0.001; 3-hydroxykynurenine, *r* 0.44, *P* < 0.001). Increased urinary levels of neutral amino acids and imino acid proline were also common in COVID-19, suggesting specific transport defects. Urine metabolomics identified major alterations in the tryptophan-kynurenine pathway, consistent with changes in host metabolism during SARS-CoV-2 infection. The association between increased urinary levels of kynurenines, inflammation and COVID-19 severity supports further evaluation of these easily available biomarkers.

## Introduction

The official global death toll from the coronavirus disease 2019 (COVID-19) pandemic has reached 5.5 million by January 2022, but the exact number has been estimated between two and four times that number of excess deaths. While SARS-CoV-2 has direct cytotoxic effects, the host response contributes to the multi-organ damage and fatal outcome associated with COVID-19. The role of an exaggerated host response in the poor outcomes is supported by the beneficial effects of interventions targeting the immune system such as dexamethasone, baricitinib and tocilizumab^1–4^. Despite these major advances, the determinants of severe COVID-19 remain poorly understood, and a better understanding of the host response to SARS-CoV-2 infection is needed.

The link between host metabolism and immune response is well established and bidirectional. Metabolites regulate immune responses and, conversely, immune stimulation elicits metabolic reprogramming in cells. Since the onset of the COVID-19 pandemic, the few studies that examined metabolic signatures in the serum of patients with COVID-19 consistently observed metabolic alterations associated with the severity of the immune response in patients with SARS-CoV-2 infection^5–8^.

Because it is an easily and non-invasively available biological fluid, the urine is becoming an important source for disease biomarker discovery. Urine abnormalities are common during SARS-CoV-2 infection, including low-molecular weight proteinuria, inappropriate uricosuria and neutral aminoaciduria, some of these features being associated with disease severity and outcome^9–11^.

In a proof-of-concept study, we performed quantitative urine metabolic profiling in consecutive, well-phenotyped patients with COVID-19 and healthy controls, to provide insights into disease mechanisms and suggest potential biomarkers of disease severity.

## Results

### Study population, COVID-19 severity and outcomes

**Figure 1:**
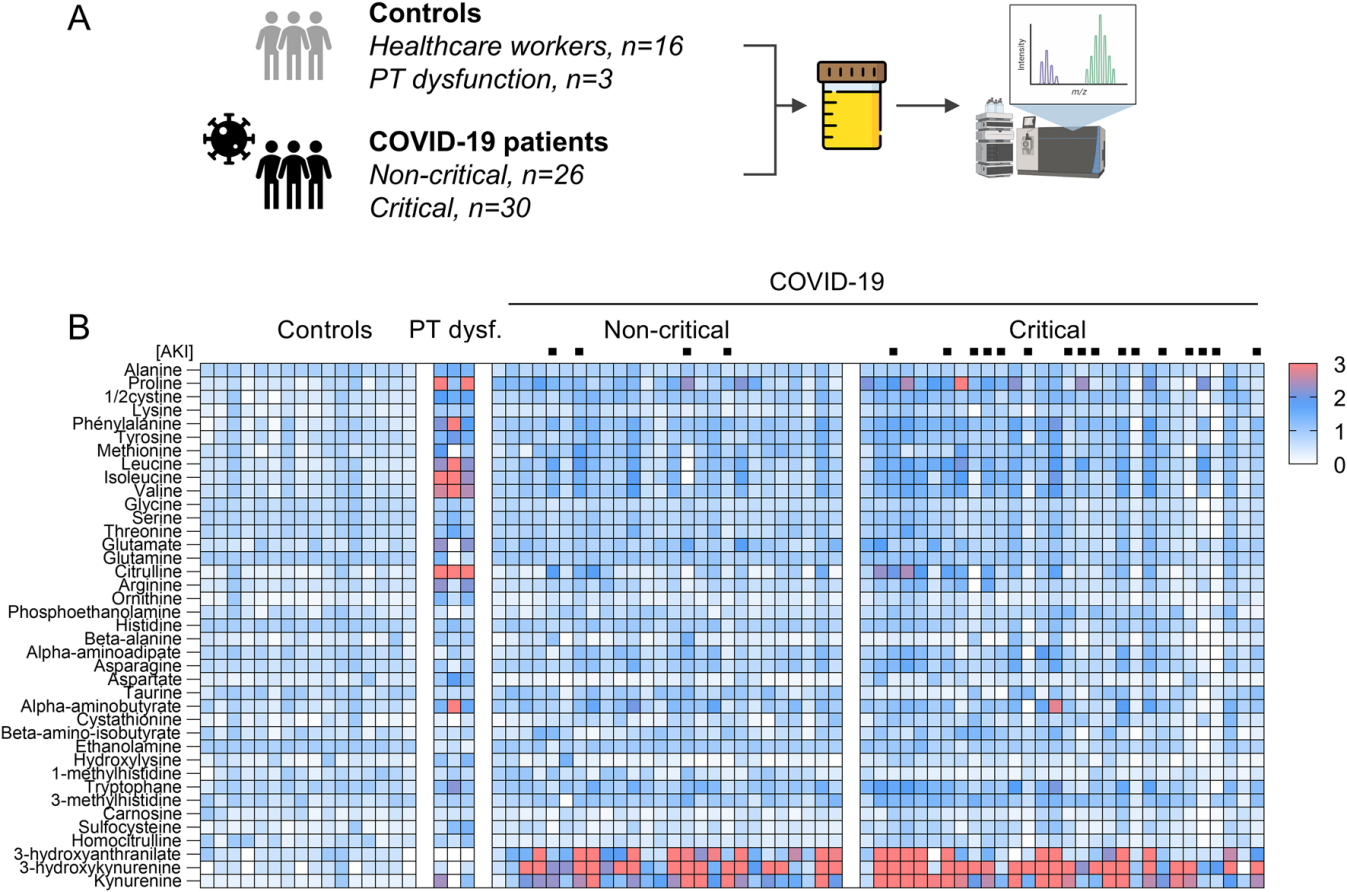
Urine Metabolomics in patients with COVID-19. (**A**) Study design. Urine samples obtained from patients with COVID-19 (n = 56, including 26 non-critical, 30 critical patients) were assessed using LC–MS/MS and compared to those obtained from healthy controls (n = 16 healthcare workers) and patients with proximal tubule (PT) dysfunction (n = 3) caused by tenofovir-related nephrotoxicity, Hartnup disease and Dent disease, respectively. Created in part with BioRender.com. (**B**) Heatmap of relative urinary concentrations of amino acids and metabolites in controls and patients hospitalized with COVID-19, stratified for disease severity. The 95th percentile of log-transformed values for each metabolite in healthy controls was considered as the reference. Patients with acute kidney injury (AKI) are identified by a black square on top of the heatmap.

To investigate potential changes in urine metabolite concentrations associated with COVID-19, we enrolled 56 patients hospitalized with SARS-CoV-2 infection and 16 healthy controls with no history of COVID-19 (Fig. 1A; Supplementary Fig. 1; Table 1).

**Table 1:** Baseline characteristics of COVID-19 patients, stratified for disease severity.

Demographics and comorbidities	COVID-19		
	Healthy controls	Non-critical	Critical
	n = 16	n = 26	n = 30
Age, median (IQR), years	49 (35–61)	54 (42–69)	64 (55–71)
Male gender—no. (%)	5 (31)	18 (69)	25 (83)
**Ethnic group—no. (%)**			
White	16 (100)	22 (85)	23 (77)
Black	0 (0)	3 (12)	4 (13)
Other	0 (0)	1 (4)	3 (10)
Ischemic heart disease—no. (%)	0 (0)	1 (4)	4 (14)
Chronic kidney disease—no. (%)	1 (6)	1 (4)	5 (17)
Hypertension—no. (%)	1 (6)	6 (23)	19 (63)
Diabetes—no. (%)	0 (0)	7 (27)	12 (40)
Obesity—no. (%)	1 (6)	10/16 (63)	12/28 (43)
**Admission**			
Wave of COVID-19			
First	–	7 (27)	12 (40)
Second	–	19 (73)	18 (60)
Symptoms at admission			
Fever—no. (%)	–	19/25 (76)	22/29 (76)
Cough—no. (%)	–	21 (81)	20 (67)
Dyspnea—no. (%)	–	19 (73)	20 (67)
Pharyngeal pain—no. (%)	–	6 (23)	1 (3)
Confusion—no. (%)	–	1 (4)	4 (13)
Anosmia/ageusia—no. (%)	–	8 (31)	5 (17)
Rhinitis—no. (%)	–	5 (19)	2 (7)
Diarrhea—no. (%)	–	8 (31)	7 (23)
Chest pain—no. (%)	–	5 (19)	2 (7)
Oxygen saturation, median (IQR), %	–	92 (89–93)	86 (75–92)
CRP, median (IQR), mg/l	–	97 (76–136)	102 (56–145)
eGFR, median (IQR), ml/min per 1.73 m^2^	–	90 (66–100)	73 (46–92)
LDH, median (IQR), IU/l	–	349 (303–428)	424 (289–556)
Lymphocytes, median (IQR), µl^−1^	–	1115 (810–1350)	825 (590–1090)
**Worst biological values during hospital stay**			
Peak CRP, median (IQR), mg/l	–	103 (78–167)	321 (212–377)
Peak serum creatinine, median (IQR), mg/dl	–	1.0 (0.8–1.2)	1.2 (0.9–1.8)
Nadir lymphocyte count, median (IQR), µl^−1^	–	825 (580–1190)	315 (220–510)
Peak LDH, median (IQR), IU/l	–	410 (347–508)	638 (515–769)
Peak D-dimers, median (IQR), ng/ml	–	775 (464–1198)	2127 (812–6260)
Peak ferritin, median (IQR), µg/l	–	951 (398–1619)	1498 (831–2669)
**Outcome**			
Hospital LOS, median (IQR), days	–	6 (5–10)	33 (17–96)
Death—no. (%)	–	0 (0)	14 (47)
Mechanical ventilation—no. (%)	–	0 (0)	25 (83)
AKI—no. (%)	–	4 (15)	16 (53)
AKI requiring dialysis—no. (%)	–	0 (0)	7 (23)

Continuous variables are expressed as median and interquartile range (IQR), and categorical variables as numbers (no.) and percentages (%). *CRP* C-reactive protein; *eGFR (CKD-EPI)* estimated glomerular filtration rate derived from serum creatinine level using the Chronic Kidney Disease Epidemiology Collaboration equation; *LDH* lactate dehydrogenase; LOS, length of stay; *AKI* acute kidney injury.

Baseline characteristics of patients with COVID-19 are provided in Table 1. Median age (IQR) at the time of admission was 62 years (47–69); 77% were males; and 45%, 34% and 50% had hypertension, diabetes or were obese, respectively. According to the WHO classification, 30 patients had critical and 26 non-critical COVID-19. Patients classified as critical tended to be older (64 [55–71] vs. 54 years [42–69]) and had lower oxygen saturation upon admission (86% [75–92] vs. 92% [89–93]). Patients with critical COVID-19 also had higher peak of plasma C-reactive protein (CRP) during hospitalization (321 [212–377] vs. 103 [78–167] mg/l), longer hospital stays (33 [17–96] vs. 6 [5–10] days) and higher rates of death (14/30 [47%] vs. 0/26 [0%]), need for mechanical ventilation (25/30 [83%] vs. 0/26 [0%]), and acute kidney injury (16/30 [53%] vs. 4/26 [15%]) as compared with non-critical patients (Table 1). Urine specimens were collected a median of 4 days (2–11) after admission; the delay between symptom onset and sampling was similar in critical and non-critical patients (13 days [10–22] vs. 13 [10–16] days in critical and non-critical COVID-19 patients, respectively). No difference in the use of dexamethasone or hydroxychloroquine nor in serum creatinine levels was observed between critical and non-critical patients at the time of sampling (Supplementary Table 2). After a median of 16 days (6–36), 28 (50%) patients were discharged without mechanical ventilation, 25 (45%) progressed to respiratory failure requiring mechanical ventilation, and 3 (5%) died without mechanical ventilation (Supplementary Fig. 1). Of those requiring mechanical ventilation, 11 (44%) died and 14 (56%) survived and were discharged.

### Dysregulation of the tryptophan-kynurenine pathway in patients with COVID-19

**Figure 2:**
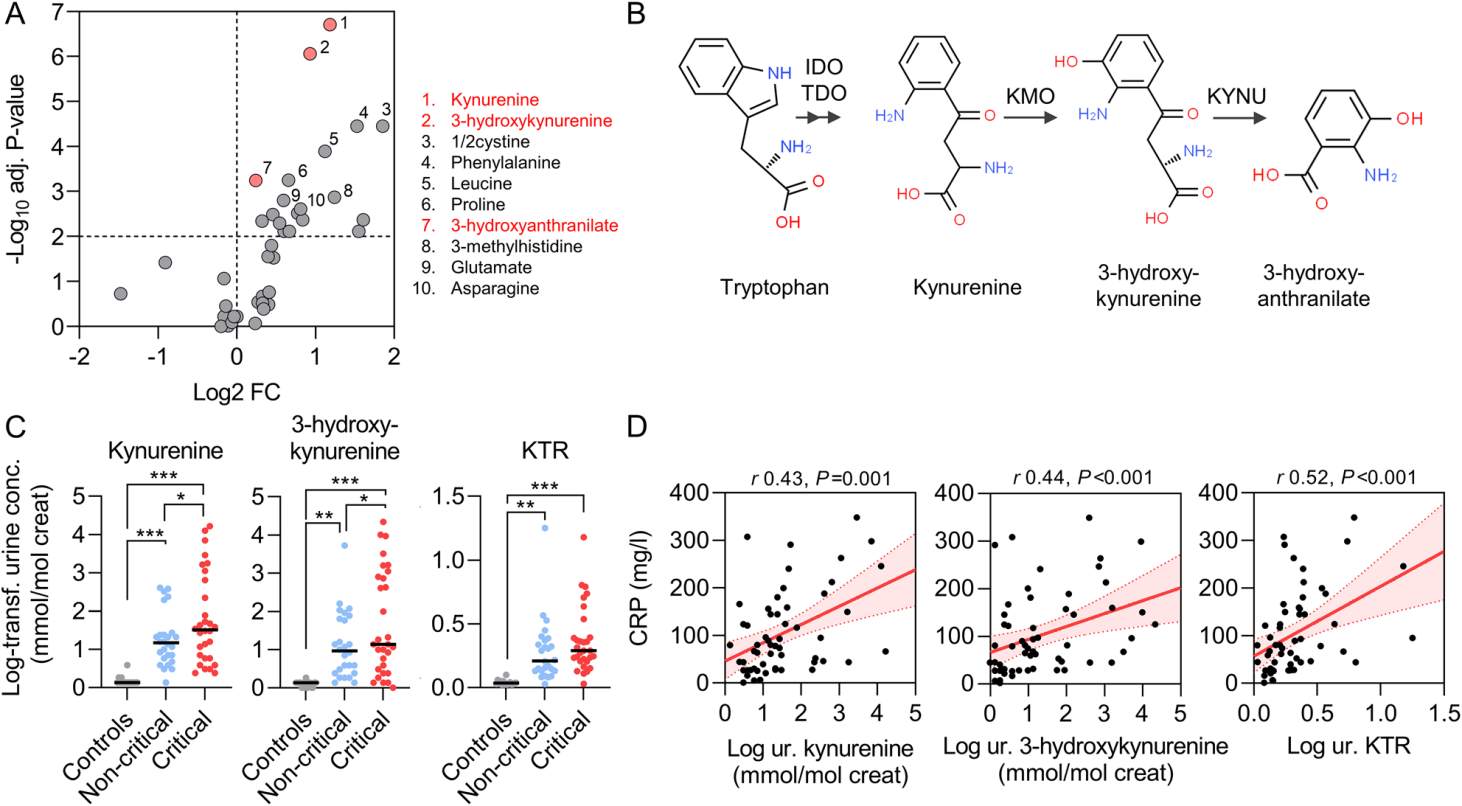
Dysregulation of the tryptophan-kynurenine pathway in the urine of patients with COVID-19. (**A**) Volcano plot comparing log-transformed levels of urine metabolites in COVID-19 patients versus healthy controls. Significantly altered (increased) tryptophan metabolites are highlighted in red and the ten top dysregulated metabolites are identified with numbers and text. Two-sided Mann–Whitney U test followed by Benjamini and Hochberg multiple comparison test with false discovery rate (FDR) < 0.01. (**B**) Metabolites of the kynurenine pathway. Chemical structures were obtained from ChemSpider (www.chemspider.com), 2021. TDO, tryptophan 2,3-dioxygenase; IDO, indole 2,3-dioxygenase; KMO, kynurenine 3-monooxygenase; KYNU, kynureninase. (**C**) Comparison of log-transformed urinary levels of kynurenines and kynurenine to tryptophan ratio among controls (grey), non-critical (blue) and critical (red) COVID-19 patients. Data are individual values and medians. Comparisons using a one-way ANOVA followed by Holm-Sidak correction for multiple comparison. (**D)** Systemic inflammation, assessed by the plasma level of C-reactive protein (CRP) at the time of sampling, correlates with urinary (ur.) concentration of kynurenine, 3-hydroxykynurenine and the kynurenine to tryptophan ratio (KTR) in COVID-19. Data are individual values (black dots), and linear regressions with 95% confidence intervals (red lines).

In order to unravel specific disturbances in amino acid metabolism pathways associated with COVID-19 and disease severity, we used a targeted metabolomic approach to quantify changes in the urine from patients with COVID-19 vs. healthy controls. The heatmap and volcano plot showed major increases in urinary concentrations of tryptophan metabolites, including kynurenine, 3-hydroxykynurenine and 3-hydroxyanthranilate, along with less marked yet significant increases in urinary levels of tryptophan and other amino acids in patients with SARS-CoV-2 infection as compared with controls (Fig. 1B and 2A, Supplementary Fig. 2; Supplementary Table 3). Because of the expected unbalanced age and gender distribution in COVID-19 vs. controls, in line with previous studies^8^, logistic regressions adjusted for age and gender validated the independent association between COVID-19 and increased urinary levels of metabolites of the kynurenine pathway (Fig. 2B; Supplementary Table 4). Unadjusted regressions provided similar results (Supplementary Table 4). As a result, the kynurenine to tryptophan ratio, a reliable indicator of activation of the kynurenine pathway of tryptophan degradation^6^, was drastically increased among patients with COVID-19 as compared with healthy controls. Such activation of the kynurenine pathway was not observed in three control patients with kidney proximal tubule dysfunction unrelated to SARS-CoV-2 infection (Fig. 1B).

### Urine levels of kynurenines, systemic inflammation and severity of COVID-19

Among patients with COVID-19, the level of these urinary metabolites was also associated with disease severity, and patients with critical COVID-19 had higher levels of urinary tryptophan metabolites than those with non-critical disease (Fig. 2C). Systemic inflammation assessed by CRP levels at the time of sampling was tightly correlated with urinary concentration of kynurenine (Spearman, *r* 0.43, *P* = 0.001) and 3-hydroxykynurenine (*r* 0.44, *P* < 0.001), as well as with the kynurenine to tryptophan ratio (*r* 0.52, *P* < 0.001) (Fig. 2D; Supplementary Table 2). The association between urinary levels of kynurenines and COVID-19, and the correlation with systemic inflammation were observed both in patients who did not receive dexamethasone (first wave of the pandemic) and in those treated with steroids (Supplementary Fig. 3). Moreover, in this small cohort of patients hospitalized with COVID-19, higher urinary levels of kynurenine were associated with the composite of death or mechanical ventilation (unadjusted OR 2.01, 95% CI 1.08–3.71, *P* = 0.03), independently from baseline kidney function and occurrence of acute kidney injury (adjusted OR 2.28, 95% CI 1.15–4.53, *P* = 0.02). We found no association between urinary levels of kynurenines and acute kidney injury nor with serum creatinine at the time of sampling (Supplementary Fig. 4; Supplementary Table 5).

### Aminoaciduria in COVID-19

**Figure 3:**
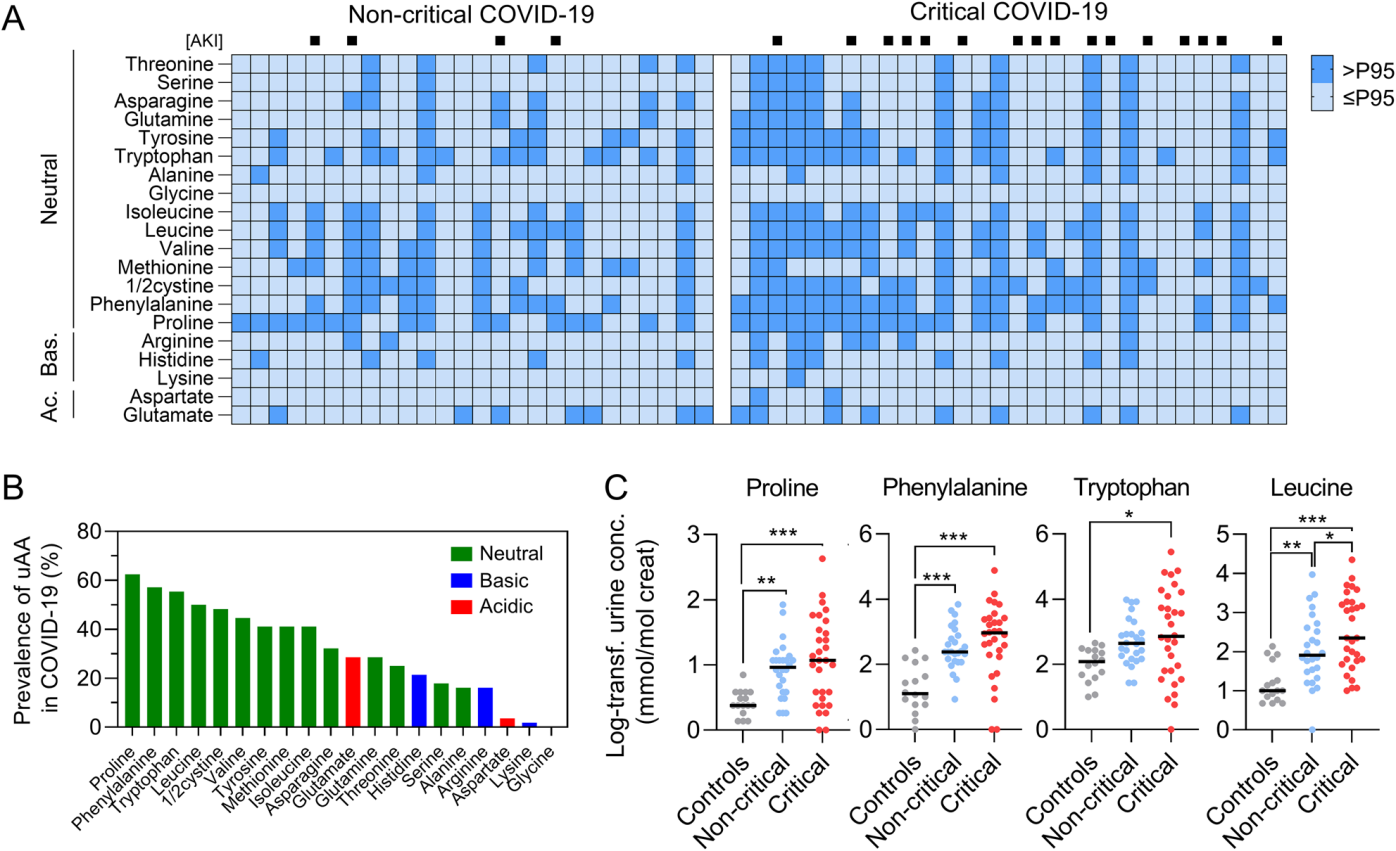
Prevalence and pattern of aminoaciduria in COVID-19. (**A**) Heatmap of aminoaciduria in COVID-19 patients. Presence (dark blue) of aminoaciduria was defined as a concentration above the 95th percentile of log-transformed values in healthy controls. Dark blue (vs. light blue) cells indicate presence (vs. absence) of aminoaciduria, for the 20 classical amino acids. Patients with acute kidney injury (AKI) are identified by a black square on top of the heatmap. Bas., basic; Ac., acidic. (**B**) Prevalence of aminoaciduria in COVID-19. Neutral, basic and acidic amino acids are indicated in green, blue and red, respectively. (**C**) Comparison of log-transformed urinary levels of the most prevalent amino acids among controls (grey), non-critical (blue) and critical (red) COVID-19 patients. Data are individual values and medians. Comparisons using a one-way ANOVA followed by Holm-Sidak correction for multiple comparison.

In addition to increased levels of kynurenines, metabolomic analysis showed that aminoaciduria is common in patients with COVID-19 (Fig. 3). More than two thirds of COVID-19 patients showed increased levels of two or more amino acids in the urine (*P* < 0.001 vs. healthy controls), mainly including neutral amino acids (i.e. phenylalanine, tryptophan and leucine, 50–60%) and the imino acid proline (63%) (Fig. 3; Supplementary Tables 3 and 6)^9^.

## Discussion

In this proof-of-concept study, we provide preliminary evidence that quantitative urine metabolite profiling is a reliable tool to detect major alterations in the tryptophan-kynurenine pathway in patients with COVID-19. These metabolic disturbances correlated with systemic inflammation and were associated with disease severity. We also demonstrated that aminoaciduria is highly common during SARS-CoV-2 infection, mainly including neutral amino acids (i.e. phenylalanine, tryptophan and leucine) and the imino acid proline.

The kynurenine pathway represents the major route (95%) for the degradation of the essential amino acid tryptophan and generates metabolites known as kynurenines. The kynurenines are involved in immunity, inflammatory response and neurotransmission, and intermediates in these pathways are also used to synthesize nicotinamide adenine dinucleotide, which plays a critical role in generating cellular energy^12^. Tryptophan 2,3-dioxygenase and indole 2,3-dioxygenase (IDO, isoforms 1 and 2) catalyze the first, rate-limiting step of the pathway (Fig. 2B). In immune cells, as in most extrahepatic tissues, the kynurenine pathway is initiated by IDO, where its expression is induced by interferon-γ, tumor necrosis factor α, and pathogenic infections, such as influenza A virus or SARS-CoV-2 infection^5–8,13^. As a result, elevated serum levels of kynurenine have been observed in humans in a number of diseases and after interferon treatment, and the enzyme is induced in rodents after administration of interferon inducers or influenza virus^12–14^. In turn, IDO regulates immune responses by depleting tryptophan and generating kynurenines which activate the aryl hydrocarbon receptor and suppress the activity of natural killer cells, dendritic cells, monocytes and macrophages; block T cell proliferation; induce T cell death; and promote proliferation of regulatory T cells^12^. Although kynurenines may help regulating immune responses, they also contribute to evasion from antiviral immunity, thereby promoting viral replication and organ damage^15,16^. Interestingly, modulation of tryptophan metabolism with epacadostat, an IDO inhibitor, successfully suppressed SARS-CoV-2-induced proinflammatory cytokine release in experimental models, supporting a direct contribution of the tryptophan-kynurenine pathway to the exaggerated host immune response and its therapeutic potential in COVID-19^7^. The detection of tryptophan metabolites in the urine is in agreement with previous metabolomic studies performed on the serum from COVID-19 patients^5–8^, and with the physicochemical characteristics of kynurenine and derivatives (i.e. low molecular weight [~ 200 Da], partial protein-binding, high water solubility) implying they are excreted via glomerular filtration^17,18^. The low urinary concentration of tryptophan metabolites in controls is in line with data from the Urine Metabolome Database^19^. A recent case–control study investigated the urine metabolome of patients hospitalized with COVID-19 and observed alterations in the tryptophan-kynurenine pathway in individuals who developed acute kidney injury, as compared to those who did not^11^. However, such dysregulation in urine tryptophan metabolites was independent from the presence of acute kidney injury among patients with severe COVID-19, suggesting an association with COVID-19 severity rather than kidney injury per se^11^. Our data provide additional evidence that urine metabolomic disturbances in the tryptophan-kynurenine pathway associate with COVID-19 severity rather than kidney dysfunction.

We also observed a significant loss of various amino acids in the urine of COVID-19 patients, validating our previous observation that SARS-CoV-2 causes proximal tubule dysfunction and aminoaciduria^9^. The main urine amino acids during COVID-19 include neutral amino acids (e.g., phenylalanine, leucine, tryptophan) and imino acids (e.g., proline), which are transported by B^0^AT1/SLC6A19 and SIT1/SLC6A20, respectively^20,21^. Mutations in *SLC6A19* cause a defective neutral amino acid transport in the kidney proximal tubule and the small intestine, a rare condition characterized by neutral aminoaciduria and known as Hartnup disease^20^. The mechanism of aminoaciduria during SARS-CoV-2 infection remains unknown but it is interesting to note that angiotensin-converting enzyme 2 (ACE2), the cellular receptor mediating virus entry into host cells (through interaction with SARS-CoV-2 spike protein), heterodimerizes with B^0^AT1/SLC6A19 or SIT1/SLC6A20 to enable membrane expression of these transporters in the intestinal or kidney epithelium^22–24^. Interestingly, genomewide association study of severe COVID-19 identified a genetic susceptibility locus on 3p21.31 containing 6 genes, and recent data suggested *SLC6A20* as one of the most likely gene candidates accounting for the association at this locus^25,26^. Mechanistic studies will be needed to identify molecular alterations and transport defects causing the observed changes in urine metabolite profile of patients with COVID-19. The urinary loss of other neutral amino acids suggested that altered amino acid absorption by the kidney proximal tubule contributes to the modest albeit significant increase in urinary levels of tryptophan, despite reduced serum or plasma levels^5–8^, during SARS-CoV-2 infection. Of note, the combination of proximal tubule dysfunction and systemic activation of the kynurenine pathway resulted in an increase in urinary tryptophan levels that was less important than that observed for other neutral amino acids, such as phenylalanine and leucine.

The strengths of our study include a well-phenotyped cohort of non-critical and critical COVID-19 patients; accurate quantitative analysis of an extensive amino acid profile; and availability of urine specimen collected early during hospitalization. We also acknowledge limitations, including the limited sample size and single center design; the lack of a control group with hospitalized patients unrelated to COVID-19 (i.e. with influenza A infection or sepsis) and/or control patients receiving similar treatments; the absence of untargeted metabolomic analysis on the urine and concurrent serum analysis, and the lack of longitudinal follow-up of urine metabolites concentrations. Other metabolites of the tryptophan-kynurenine pathway, such as quinolinate and kynurenic acid are not routinely quantified on our platform and were not available in this study. Although our observations are consistent with previous findings on the serum, future studies will need to validate the usefulness of urine metabolomics in patients with COVID-19.

In summary, urine metabolite profiling identifies major alterations in the tryptophan-kynurenine pathway, consistent with changes in host metabolism during SARS-CoV-2 infection. Among COVID-19 patients, increased urine levels of kynurenine and metabolites are associated with systemic inflammation and disease severity, suggesting their potential value as biomarkers and/or therapeutic target.

## Methods

### Study design and participants

Urine samples were collected from consecutive adult patients admitted to the Cliniques universitaires Saint- Luc, Brussels, Belgium with a diagnosis of SARS-CoV-2 infection during the first (February 23, 2020 to April 18, 2020) and second (September 21, 2020 to November 21, 2020) waves of the pandemic. Patients with kidney failure (chronic dialysis or kidney transplantation) were excluded. COVID-19 diagnosis was based on the detection of SARS-CoV-2 by real-time reverse transcription polymerase chain reaction on nasopharyngeal swab or broncho-alveolar lavage.

Classification of disease severity was based on World Health Organization ‘COVID-19 Clinical management: living guidance’ (Jan 25, 2021). Mild disease was defined as COVID-19 without evidence of viral pneumonia or hypoxia; moderate disease, as clinical signs of pneumonia (fever, cough, dyspnea, fast breathing) but no signs of severe pneumonia, including oxygen saturation ≥ 90% on room air; severe disease, as clinical signs of pneumonia plus respiratory rate > 30 breaths/min, severe respiratory distress, or oxygen saturation < 90% on room air; and critical COVID-19, as respiratory failure requiring mechanical ventilation, shock and/or other organ failure requiring intensive care unit admission.

The standard treatment for patients hospitalized for COVID-19 during the first wave included hydroxychloroquine 400 mg b.i.d. on the first hospital day then 200 mg b.i.d. for four additional days in patients without contraindication, as recommended at that time by the Belgian COVID-19 interim guidelines. The standard treatment for severe and critical patients hospitalized during the second wave consisted of oral dexamethasone at a dose of 6 mg once daily for up to 10 days. None of the patients received tocilizumab or baricitinib. Patients were followed until death or end of study follow-up (July 01, 2021).

Non-infected healthcare workers were used as healthy controls. Stored urine samples from patients with kidney proximal tubule dysfunction unrelated to COVID-19 (tenofovir disoproxil fumarate-related nephrotoxicity, Hartnup disease and Dent disease) were used as positive controls as impaired tubular transport causes aminoaciduria, as part of the renal Fanconi syndrome.

Urine samples were either collected in the setting of the prospective COBISA study (‘Collecting biological samples of recovering patients to better understand COVID-19’) after informed consent has been obtained, or as residual material following the completion of routine clinical laboratory testing. All samples were stored at − 80 °C. The definition of acute kidney injury was adapted from the KDIGO guidelines and restricted to changes in serum creatinine, as previously done^11^, as urine output was not digitally recorded in most patients (i.e. those admitted to a general ward).

The study was conducted in accordance with the World Medical Association’s Declaration of Helsinki, the Belgian law related to experiments in humans dated May 7, 2004, the General Data Protection Regulation 2016/679 and the Belgian law of July 30, 2018 regarding the protection of personal data, and was approved by the Ethical Review Board of Cliniques universitaires Saint-Luc/UCLouvain (protocol ID: 2020/26NOV/586).

### Targeted metabolomics analysis by LC–MS/MS

Liquid chromatography/tandem mass spectrometry (LC–MS/MS) accurate quantification of 39 urinary amino acids and related metabolites was performed and adapted using the Waters™ Kairos™ Amino Acid Kit in the Laboratory of Inherited Metabolic Diseases/Biochemical Genetics (Cliniques universitaires Saint-Luc, UCLouvain, Brussels). The technique is mainly used in clinical practice for the screening and follow-up of inborn errors of metabolism, and therefore targets amino acids and related compounds relevant to this purpose. Prior to LC–MS/MS analysis, proteins were precipitated using a sulfosalicylic acid solution containing stable-isotope labeled internal standards. Subsequently, the compounds containing primary and secondary amines were derivatized in a reaction with 6-aminoquinolyl-N-hydroxysuccinimidyl carbamate (AccQTag™ Reagent). The diluted derivatized samples were injected and eluted by reverse phase liquid chromatography using a column (CORTECS C18 UPLC, 2.1 × 150 mm, particle size 1.6 μm, Waters ref 186,007,096) heated at 60 °C on an Acquity UPLC-I-Class system (Waters corporation, Wexford, Ireland). The mobile phases A [Water, 0.1% formic acid] and B [Acetonitrile, 0.1% formic acid] at a flow rate of 0.45 mL/min were used at the following concentrations and linear gradients: 0–1 min: 1% B; increase of B to 6% over 1 min; increase of B to 9.5% over 3 min; increase of B to 10.5% over 7.5 min; increase of B to 95% over 1 min; 95% B from 13.5 to 14.2 min; decrease of B to 1% over 0.1 min and 1% B from 14.3 to 16 min. Multiple Reaction Monitoring (MRM) analyses were performed using a Xevo™ TQ-S micro mass spectrometer (Waters corporation, Wexford, Ireland) in positive electrospray ionization (ESI). The ESI spray voltage was 2000 V. The cone gas flow rate was 20 L/h, desolvation gas flow 1000 L/h and desolvation temperature 500 °C. Quantification was performed with appropriate calibration curves using ratios between area under the curve of the compound and its specified stable-isotope labeled internal standard. Results were normalized to creatinine content, as previously described^11^. Mass spectrometry settings for each analyte are summarized in Table S1.

### Statistical analyses

Results are presented as medians and interquartile ranges (IQR) for continuous variables and as numbers and proportions for categorical variables. Concentrations of urinary metabolites were log-transformed using the following formula: x = log2(x + 1−min(x)) to obtain normal or near-normal distributions of urine metabolites. The upper limit of normal metabolite abundance range was defined for each metabolite as the 95^th^ percentile of the log-transformed urinary concentration in healthy controls. Other continuous variables were expressed in their natural units without standardization.

Comparisons between groups were performed using unpaired t-test, Mann–Whitney U test, Kruskal–Wallis, or χ^2^ test, as appropriate. Volcano plot was performed using a two-sided Mann–Whitney U test followed by Benjamini and Hochberg multiple comparison test with false discovery rate (FDR) < 0.01 and fold changes based on median values of untransformed metabolite urinary concentrations. Logistic regressions were used to assess association with COVID-19 and outcome; the Spearman test, to assess correlations; and Cox proportional hazard regressions, for time to event analyses (where events were defined as the need for invasive mechanical ventilation or death).

Statistical analyses were performed using GraphPad Prism (version 9.0), Stata (version 16.0) and R (version R-4.1.21) software. Unless specified otherwise, all tests were two-tailed and a *P* value < 0.05 was considered significant.

## Supplementary Information

Supplementary Information.

## Data Availability

The raw datasets generated on the LC–MS/MS instrument during the current study are available from the corresponding author on reasonable request.
