# Supplementary material for: Urine metabolomics links dysregulation of the tryptophan-kynurenine pathway to inflammation and severity of COVID-19
Source: Sci Rep. 2022 Jun 15;12:9959. doi: 10.1038/s41598-022-14292-w (PMC9198612; doi:10.1038/s41598-022-14292-w)
Supplement: Supplementary file 1 — Supplementary Information. [file 41598_2022_14292_MOESM1_ESM.docx]

***Supplementary information***

**Urine Metabolomics Links Dysregulation of the Tryptophan-Kynurenine Pathway to Inflammation and Severity of COVID-19:**

Joseph P. Dewulf, Manon Martin, Sandrine Marie, Fabie Oguz, Leila Belkhir, Julien De Greef, Jean Cyr Yombi, Xavier Wittebole, Pierre-François Laterre, Michel Jadoul, Laurent Gatto,6 Guido T. Bommer, and Johann Morelle

**Table of content**

**Supplementary Figure 1.** Flowchart of the study ………………………………………...…..2

**Supplementary Figure 2.** Comparisons of urinary concentrations of kynurenine pathway metabolites and the kynurenine to tryptophan ratio in patients with COVID-19 vs. healthy controls …………………………………………………………………………………………3

**Supplementary Figure 3.** Urinary concentrations of kynurenine pathway metabolites and correlation with systemic inflammation in patients treated with or without dexamethasone ….4

**Supplementary Figure 4.** Lack of association between urinary concentrations of tryptophan-kynurenine pathway metabolites and acute kidney injury …………………………………….5

**Supplementary Table 1.** Mass spectrometry settings ………………………………………...6

**Supplementary Table 2.** Characteristics of COVID-19 patients at urine sampling ……….....8

**Supplementary Table 3.** Changes in urinary metabolite concentrations in patients with COVID-19 vs. healthy controls ………………………………………………………………..9

**Supplementary Table 4.** Logistic regression analyses of urinary kynurenines concentrations in COVID-19 patients *vs.* healthy controls …………………………………………………...10

**Supplementary Table 5.** Changes in urinary metabolite concentrations in COVID-19 patients with vs. without acute kidney injury ……………………………………………………….....11

**Supplementary Table 6.** Prevalence of aminoaciduria in controls and COVID-19 patients ..12

**Supplementary Figure 1. Flowchart of the study.**

**
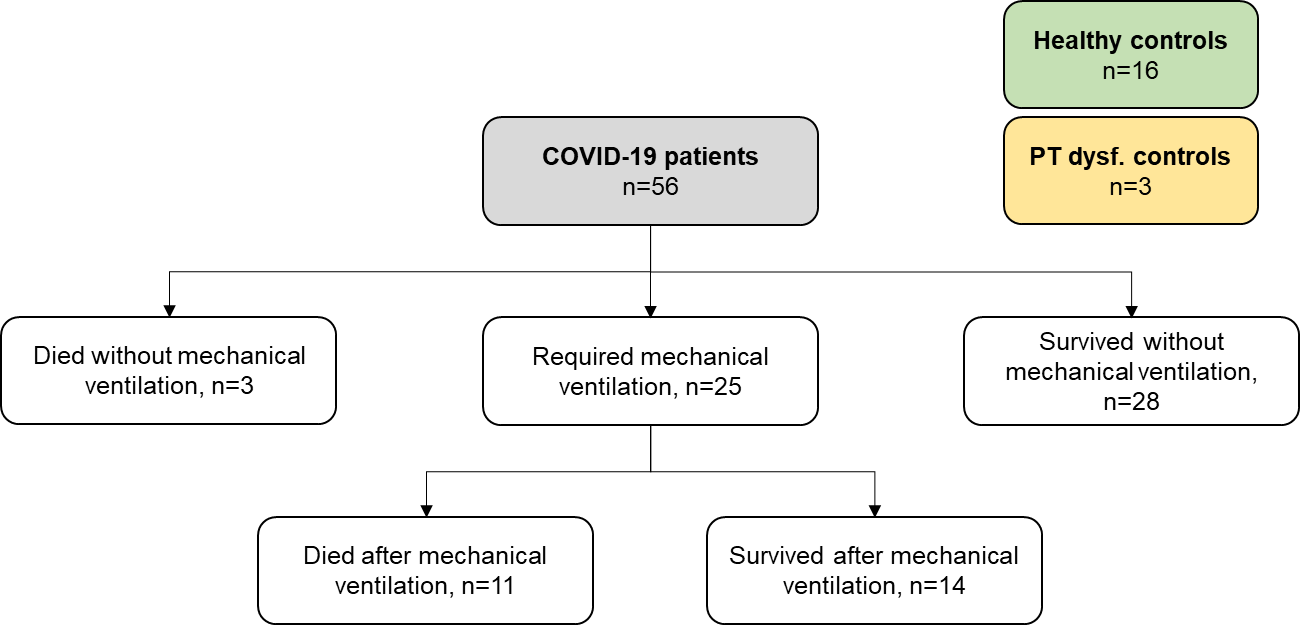
**

PT dysf., proximal tubule dysfunction.

**Supplementary Figure 2. Comparisons of urinary concentrations of kynurenine pathway metabolites and the kynurenine to tryptophan ratio in patients with COVID-19 vs. healthy controls.**

**
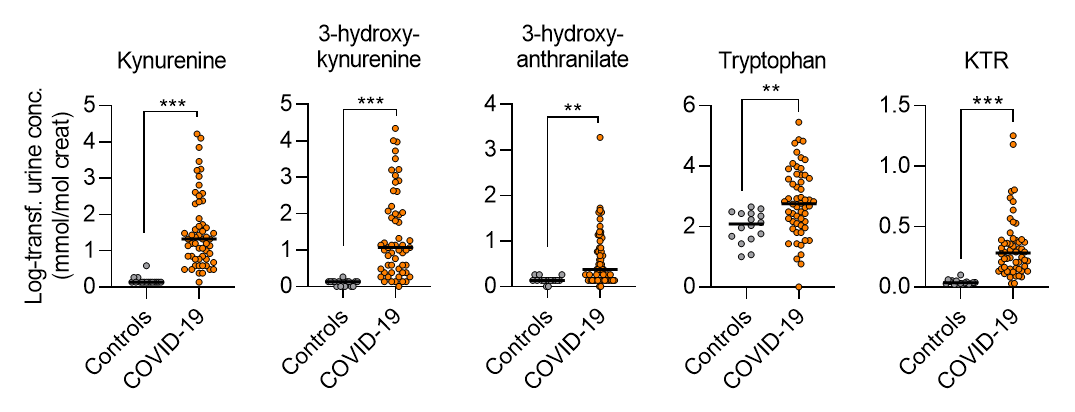
**

Urinary concentrations of kynurenine, 3-hydroxykynurenine and the kynurenine-to-tryptophan ratio (KTR) in patients with COVID-19 (orange dots) vs. healthy controls (grey dots). Data are individual values and medians. Comparisons using unpaired t-tests.

**Supplementary Figure 3. Urinary concentrations of kynurenine pathway metabolites and correlation with systemic inflammation in patients treated with or without dexamethasone.**

**
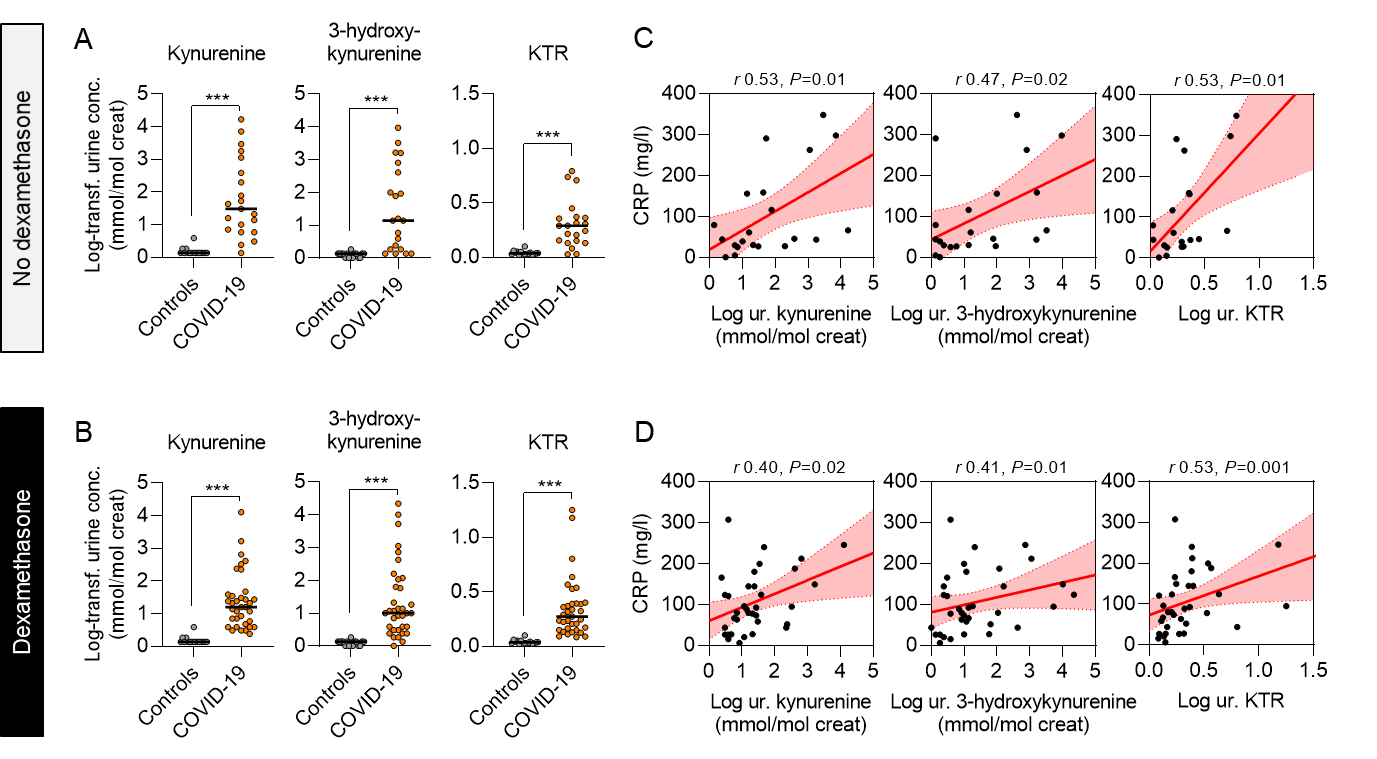
**

(**A-B**) Urinary concentrations of kynurenine, 3-hydroxykynurenine and the kynurenine-to-tryptophan ratio (KTR) in patients with COVID-19 (orange dots) not treated with (A) or treated with (B) dexamethasone vs. healthy controls (grey dots). Data are individual values and medians. Comparisons using unpaired t-tests. (**C-D**) Correlations between systemic inflammation, assessed by the plasma level of C-reactive protein (CRP) at the time of sampling, and urinary levels of kynurenine, 3-hydroxykynurenine and the KTR in COVID-19 patients not treated with (C) or treated with (D) dexamethasone.

**Supplementary Figure 4. Lack of association between urinary concentrations of tryptophan-kynurenine pathway metabolites and acute kidney injury.**

**
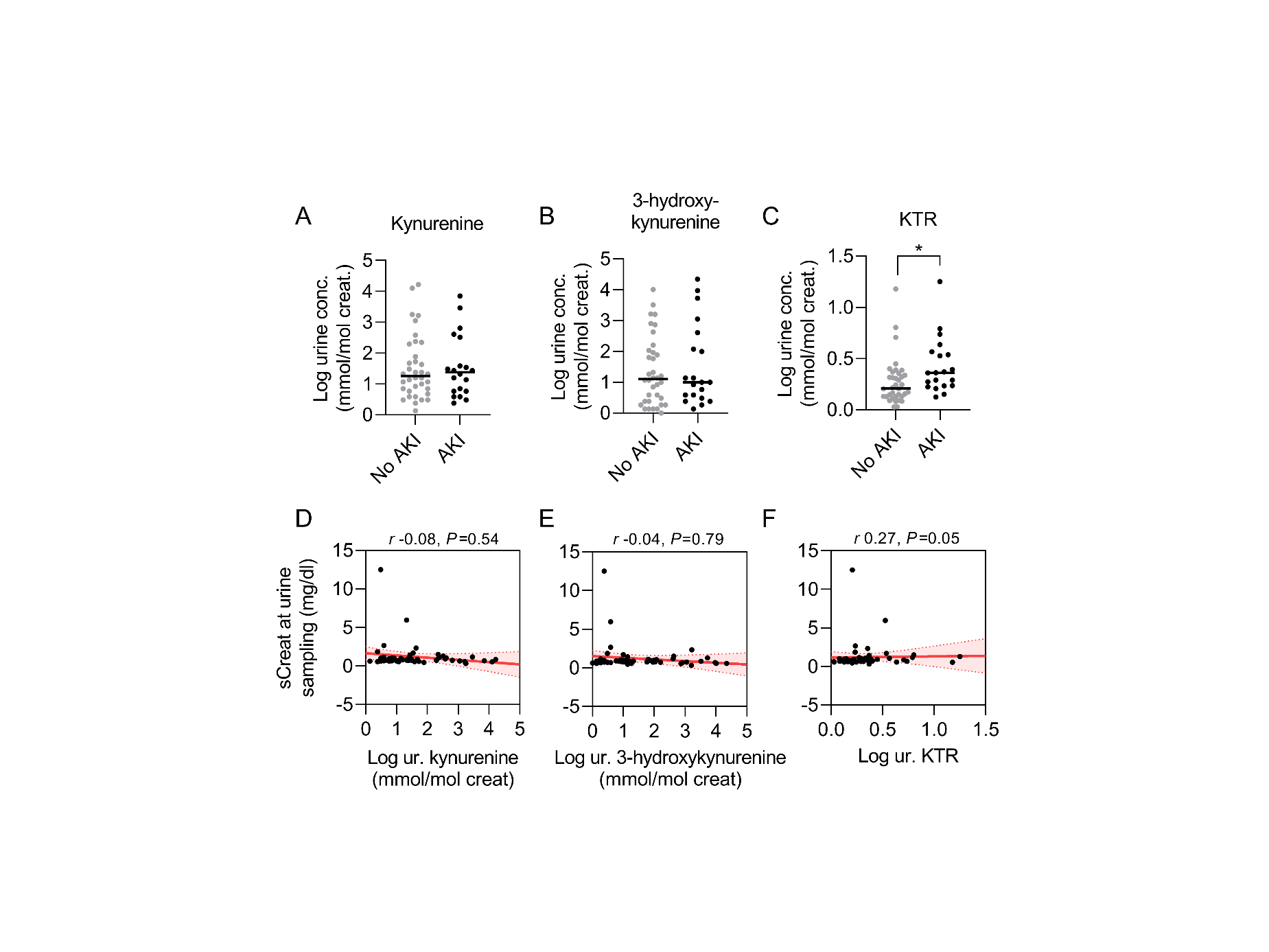
**

(**A-C**) Urinary concentrations of kynurenine, 3-hydroxykynurenine and the kynurenine-to-tryptophan ratio (KTR) in COVID-19 patients who developed (black dots) or not (grey dots) acute kidney injury (AKI) during hospitalization. Data are individual values and medians. (**D-F**) Absence of correlation between serum creatinine level at the time of sampling, and urinary levels of kynurenine, 3-hydroxykynurenine and the KTR in COVID-19 patients.

**Supplementary Table 1. Mass spectrometry settings.**

| **Compound** | **MRM transition** | **Collision energy (eV)** | **Retention time (min)** | **Internal standard** |
| --- | --- | --- | --- | --- |
| Alanine | 260.2 > 171.0 | 20 | 4.1 | Alanine ^13^C_3_, ^15^N |
| Proline | 286.2 > 171.0 | 20 | 4.7 | Proline ^13^C_5_, ^15^N |
| Cystine | 291.1 > 171.0 | 15 | 6.2 | Cystine ^13^C_6_, ^15^N_2_ |
| Lysine | 244.1 > 171.0 | 15 | 6.2 | Lysine ^13^C_6_,^15^N_2_ |
| Phenylalanine | 336.2 > 171.0 | 20 | 13.3 | Phenylalanine ^13^C_9_, ^15^N |
| Tyrosine | 352.2 > 171.0 | 20 | 6.5 | Tyrosine ^13^C_9_, ^15^N |
| Methionine | 320.2 > 171.0 | 20 | 6.9 | Methionine ^13^C_5_, ^15^N |
| Leucine | 302.2 > 171.0 | 20 | 12.3 | Leucine ^13^C_6_, ^15^N |
| Isoleucine | 302.2 > 171.0 | 20 | 11.7 | Isoleucine ^13^C_6_, ^15^N |
| Valine | 288.2 > 171.0 | 20 | 7.3 | Valine ^13^C_5_, ^15^N |
| Glycine | 246.2 > 171.0 | 20 | 3.2 | Glycine ^13^C_2_,^15^N |
| Serine | 276.2 > 171.0 | 20 | 3.1 | Serine ^13^C_3_, ^15^N |
| Threonine | 290.2 > 171.0 | 20 | 3.8 | Threonine ^13^C_4_, ^15^N |
| Glutamate | 318.2 > 171.0 | 20 | 3.5 | Glutamate ^13^C_5_,^15^N |
| Glutamine | 317.2 > 171.0 | 20 | 3.0 | Glutamine ^13^C_5_, ^15^N_2_ |
| Citrulline | 346.2 > 171.0 | 20 | 3.5 | Glutamate ^13^C_5_,^15^N |
| Arginine | 345.2 > 171.0 | 20 | 2.8 | Arginine ^13^C_6_,^15^N_4_ |
| Ornithine | 237.1 > 171.0 | 15 | 5.3 | Proline ^13^C_5_, ^15^N |
| Phosphoethanolamine | 312.2 > 171.0 | 15 | 2.8 | Aspartate ^13^C_4_, ^15^N |
| Histidine | 326.2 > 171.0 | 20 | 1.9 | Histidine ^13^C_6_, ^15^N_3_ |
| Beta-alanine | 260.2 > 171.0 | 20 | 3.7 | Glutamate ^13^C_5_,^15^N |
| Alpha-aminoadipate | 332.2 > 171.0 | 20 | 4.2 | Alanine ^13^C_3_, ^15^N |
| Asparagine | 303.2 > 171.0 | 20 | 2.5 | Asparagine ^13^C_4_, ^15^N_2_ |
| Aspartate | 304.2 > 171.0 | 20 | 3.3 | Aspartate ^13^C_4_, ^15^N |
| Taurine | 296.2 > 171.0 | 20 | 3.0 | Serine ^13^C_3_, ^15^N |
| Alpha-aminobutyrate | 274.2 > 171.0 | 20 | 5.4 | Proline ^13^C_5_, ^15^N |
| Cystathionine | 282.1 > 171.0 | 15 | 5.4 | Proline ^13^C_5_, ^15^N |
| Beta-amino-isobutyrate | 274.2 > 171.0 | 20 | 4.6 | Proline ^13^C_5_, ^15^N |
| Ethanolamine | 232.2 > 171.0 | 20 | 3.3 | Glycine ^13^C_2_,^15^N |
| Hydroxylysine | 252.2 > 171.0 | 15 | 4.6 | Histidine ^13^C_6_, ^15^N_3_ |
| 1-methylhistidine | 340.2 > 171.0 | 20 | 2.3 | Histidine ^13^C_6_, ^15^N_3_ |
| Tryptophan | 375.2 > 171.0 | 20 | 14.1 | Tryptophan ^13^C_11_, ^15^N_2_ |
| 3-methylhistidine | 340.2 > 171.0 | 20 | 2.6 | Arginine ^13^C_6_,^15^N_4_ |
| Carnosine | 397.2 > 171.0 | 25 | 3.0 | Histidine ^13^C_6_, ^15^N_3_ |
| Sulfocysteine | 372.2 > 171.0 | 20 | 3.1 | Serine ^13^C_3_, ^15^N |
| Homocitrulline | 360.2 > 171.0 | 20 | 4.1 | Alanine ^13^C_3_, ^15^N |
| 3-hydroxyanthranilate | 324.0 > 171.0 | 25 | 7.5 | Valine ^13^C_5_, ^15^N |
| 3-hydroxykynurenine | 395.0 > 171.0 | 25 | 7.1 | Valine ^13^C_5_, ^15^N |
| Kynurenine | 379.2 > 171.0 | 25 | 11.5 | Isoleucine ^13^C_6_, ^15^N |
| Glycine ^13^C_2_,^15^N | 249.2 > 171.0 | 20 | 3.3 | / |
| Lysine ^13^C_6_,^15^N_2_ | 248.1 > 171.0 | 15 | 6.2 | / |
| Histidine ^13^C_6_, ^15^N_3_ | 335.2 > 171.0 | 20 | 1.9 | / |
| Asparagine ^13^C_4_, ^15^N_2_ | 309.2 > 171.0 | 20 | 2.5 | / |
| Arginine ^13^C_6_,^15^N_4_ | 355.2 > 171.0 | 20 | 2.9 | / |
| Serine ^13^C_3_, ^15^N | 280.2 > 171.0 | 20 | 3.1 | / |
| Aspartate ^13^C_4_, ^15^N | 309.2 > 171.0 | 20 | 3.3 | / |
| Glutamate ^13^C_5_,^15^N | 324.2 > 171.0 | 20 | 3.5 | / |
| Threonine ^13^C_4_, ^15^N | 295.2 > 171.0 | 20 | 3.8 | / |
| Alanine ^13^C_3_, ^15^N | 264.2 > 171.0 | 20 | 4.1 | / |
| Proline ^13^C_5_, ^15^N | 292.2 > 171.0 | 20 | 4.7 | / |
| Cystine ^13^C_6_, ^15^N_2_ | 295.1 > 171.0 | 15 | 6.2 | / |
| Valine ^13^C_5_, ^15^N | 294.2 > 171.0 | 20 | 7.3 | / |
| Isoleucine ^13^C_6_, ^15^N | 309.2 > 171.0 | 20 | 11.7 | / |
| Leucine ^13^C_6_, ^15^N | 309.2 > 171.0 | 20 | 12.3 | / |
| Glutamine ^13^C_5_, ^15^N_2_ | 324.2 > 171.0 | 20 | 3.2 | / |
| Methionine ^13^C_5_, ^15^N | 326.2 > 171.0 | 20 | 6.8 | / |
| Phenylalanine ^13^C_9_, ^15^N | 346.2 > 171.0 | 20 | 13.3 | / |
| Tyrosine ^13^C_9_, ^15^N | 362.2 > 171.0 | 20 | 6.5 | / |
| Tryptophan ^13^C_11_, ^15^N_2_ | 388.2 > 171.0 | 20 | 14.2 | / |

**Supplementary Table 2. Characteristics of COVID-19 patients at urine sampling.**

| **Characteristics** | **Whole cohort n=56** | **Non-critical n=26** | **Critical n=30** | **P-value** |
| --- | --- | --- | --- | --- |
| Symptoms onset to sampling, median (IQR), days | 13 (10-19) | 13 (10-16) | 13 (10-22) | 0.31 |
| Mechanical ventilation – no. (%) | 16 (29) | 0 (0) | 16 (53) | <0.001 |
| Dexamethasone – no. (%) | 32 (57) | 14 (54) | 18 (60) | 0.64 |
| Hydroxychloroquine – no. (%) | 5 (9) | 3 (12) | 2 (7) | 0.52 |
| CRP, median (IQR), mg/l | 79 (35-153) | 49 (27-89) | 124 (59-212) | 0.008 |
| Serum creatinine, median (IQR), mg/dl | 0.8 (0.7-1.0) | 0.8 (0.7-1.0) | 0.8 (0.6-1.1) | 0.19 |
| Serum uric acid, median (IQR), mg/dl | 3.9 (3.1-5.8) | 4.2 (3.6-5.8) | 3.3 (2.4-6.3) | 0.04 |
| LDH, median (IQR), IU/l | 382 (303-496) | 354 (292-426) | 418 (349-529) | 0.05 |
| Lymphocyte count, median (IQR), µl^-1^ | 1000 (700-1640) | 1245 (860-1690) | 830 (550-1230) | 0.02 |

IQR, interquartile range; CRP, C-reactive protein level; LDH, lactate dehydrogenase.

**Supplementary Table 3. Changes in urinary metabolite concentrations in patients with COVID-19 *vs.* healthy controls.**

| **Urinary metabolite** | **Log2 FC  (log-transf. conc.)** | **Log2 FC  (non-transf. conc.)** | **Adj. P-value** |
| --- | --- | --- | --- |
| Kynurenine | 1.18 | 3.91 | <0.001 |
| 3-hydroxykynurenine | 0.93 | 3.46 | <0.001 |
| 1/2 cystine | 1.85 | 1.10 | <0.001 |
| Phenylalanine | 1.53 | 1.10 | <0.001 |
| Leucine | 1.12 | 1.08 | <0.001 |
| Proline | 0.66 | 1.05 | <0.001 |
| 3-hydroxyanthranilate | 0.24 | 1.58 | <0.001 |
| 3-methylhistidine | 1.24 | 0.59 | 0.001 |
| Glutamate | 0.60 | 1.00 | 0.002 |
| Asparagine | 0.81 | 0.71 | 0.002 |
| Tyrosine | 0.78 | 0.62 | 0.003 |
| Methionine | 0.46 | 0.89 | 0.003 |
| Valine | 0.84 | 0.70 | 0.004 |
| Cystathionine | 1.61 | 1.86 | 0.004 |
| Isoleucine | 0.32 | 0.49 | 0.005 |
| Alpha-aminobutyrate | 0.54 | 0.96 | 0.005 |
| Lysine | 1.55 | 1.30 | 0.008 |
| Ornithine | 0.60 | 0.80 | 0.008 |
| Tryptophan | 0.67 | 0.59 | 0.008 |
| Sulfocysteine | 0.44 | 1.00 | 0.02 |
| Arginine | 0.40 | 0.58 | 0.03 |
| Citrulline | 0.47 | 1.00 | 0.03 |
| Glycine | -0.91 | -0.86 | 0.04 |

FC, fold change; transf. conc., transformed concentrations; adj. adjusted. Original units: mmol/mol creatinine.

**Supplementary Table 4. Logistic regression analyses of urinary kynurenines concentrations in COVID-19 patients *vs.* healthy controls.**

|  | **Unadjusted** | | | **Adjusted*** | | |
| --- | --- | --- | --- | --- | --- | --- |
|  | **Coeff.** | **95% CI** | **P-value** | **Coeff.** | **95% CI** | **P-value** |
| Kynurenine | 12.3 | 5.0-19.5 | 0.001 | 11.9 | 4.6-19.1 | 0.001 |
| 3-hydroxykynurenine | 14.1 | 3.8-24.5 | 0.007 | 14.6 | 3.4-25.8 | 0.01 |
| 3-hydroxyanthranilate | 7.9 | 1.7-14.1 | 0.01 | 8.7 | 1.4-15.9 | 0.02 |
| Tryptophan | 0.9 | 0.2-1.5 | 0.01 | 0.7 | -0.1-1.5 | 0.10 |
| KTR | 55.7 | 21.6-89.8 | 0.001 | 56.6 | 17.3-95.9 | 0.005 |

*Adjusted for gender and age. Coeff., coefficient; 95% CI, 95% confidence interval. KTR, kynurenine to tryptophan ratio.

**Supplementary Table 5. Changes in urinary metabolite concentrations in COVID-19 patients with vs. without acute kidney injury.**

| **Urinary metabolite** | **Log2 FC** | **Adj. P-value** |
| --- | --- | --- |
| Ethanolamine | -0.27 | 0.005 |
| Tyrosine | -0.43 | 0.029 |
| Histidine | -0.36 | 0.029 |
| Tryptophan | -0.62 | 0.031 |
| Glutamate | -0.38 | 0.036 |
| Glycine | -0.22 | 0.042 |
| Threonine | -0.30 | 0.045 |
| Serine | -0.15 | 0.122 |
| Glutamine | -0.12 | 0.123 |
| Alanine | -0.18 | 0.175 |
| Asparagine | -0.27 | 0.175 |
| Alpha-aminobutyrate | -0.10 | 0.175 |
| 3-methylhistidine | 0.16 | 0.175 |
| 3-hydroxyanthranilate | -1.03 | 0.252 |
| Sulfocysteine | -0.29 | 0.258 |
| Ornithine | -0.18 | 0.261 |
| Alpha-aminoadipate | -0.12 | 0.412 |
| ½ cystine | -0.06 | 0.418 |
| Homocitrulline | -0.15 | 0.418 |
| Isoleucine | -0.10 | 0.446 |
| Arginine | -0.03 | 0.478 |
| Cystathionine | 0.23 | 0.530 |
| 1-methylhistidine | -0.43 | 0.530 |
| Lysine | -0.01 | 0.557 |
| Citrulline | -0.71 | 0.568 |
| Valine | -0.10 | 0.569 |
| Taurine | -0.34 | 0.569 |
| Carnosine | 0.00 | 0.569 |
| Aspartate | 0.18 | 0.650 |
| Beta-amino-isobutyrate | 0.00 | 0.702 |
| Methionine | 0.04 | 0.715 |
| Proline | 0.00 | 0.778 |
| Beta-alanine | -0.09 | 0.778 |
| Phosphoethanolamine | 0.06 | 0.790 |
| Hydroxylysine | -0.29 | 0.790 |
| 3-hydroxykynurenine | -0.14 | 0.790 |
| Phenylalanine | 0.11 | 0.891 |
| Leucine | -0.14 | 0.891 |
| Kynurenine | 0.12 | 0.895 |

FC, fold change; adj. adjusted. Original units: mmol/mol creatinine.

**Supplementary Table 6. Prevalence of aminoaciduria in controls and COVID-19 patients.**

|  | **Controls n=16** | **COVID-19 n=56** | **P-value** | | **Non-critical  COVID-19 n=26** | **Critical COVID-19 n=30** | | **P-value** |
| --- | --- | --- | --- | --- | --- | --- | --- | --- |
| **Patients with 0 vs. ≥1 uAA** |  |  | |  |  | |  |  |
| 0 uAA - no. (%) | 12 (75) | 5 (9) |  | | 1 (4) | 4 (13) | |  |
| ≥1 uAA - no. (%) | 4 (25) | 51 (91) | <0.001 | | 25 (96) | 26 (87) | | 0.36 |
| **Patients with 0/1 vs ≥2 uAA** |  |  | |  |  | |  |  |
| 0-1 uAA - no. (%) | 13 (81) | 12 (21) |  | | 5 (19) | 7 (23) | |  |
| ≥2 uAA - no. (%) | 3 (19) | 44 (79) | <0.001 | | 21 (81) | 23 (77) | | 0.76 |

uAA, urinary amino acid. Cut-off for the presence of aminoaciduria defined as >P95 of log-transformed values in healthy controls, considering the 20 classical amino acids. P-values were calculated using Fisher’s exact tests.
